# Extracorporeal membrane oxygenation for severe Middle East respiratory syndrome coronavirus

**DOI:** 10.1186/s13613-017-0350-x

**Published:** 2018-01-10

**Authors:** Mohammed S. Alshahrani, Anees Sindi, Fayez Alshamsi, Awad Al-Omari, Mohamed El Tahan, Bayan Alahmadi, Ahmed Zein, Naif Khatani, Fahad Al-Hameed, Sultan Alamri, Mohammed Abdelzaher, Amenah Alghamdi, Faisal Alfousan, Adel Tash, Wail Tashkandi, Rajaa Alraddadi, Kim Lewis, Mohammed Badawee, Yaseen M. Arabi, Eddy Fan, Waleed Alhazzani

**Affiliations:** 1 Department of Emergency and Critical Care, King Fahad Hospital of the University-Dammam University, PO Box 40236, Al Khobar, 31952 Saudi Arabia; 2 Department of Medicine/Intensive Care, King Abdulaziz University, Jeddah, Saudi Arabia; 3 King Abdulaziz University, Jeddah, Saudi Arabia; 4 King Abdulaziz Medical City, NGHA, Jeddah, Saudi Arabia; 5 Department of Internal Medicine, College of Medicine and Health Sciences, United Arab Emirates University, Al Ain, UAE; 6 Medical Director of Critical Care, Dr. Suliman Al-Habib Group, AlFaisal University, Riyadh, Saudi Arabia; 7 Department of Anesthesiology, Dammam University, Dammam, Saudi Arabia; 8 Department of ICU, King Fahad Hospital, Jeddah, Saudi Arabia; 9 Intensive Care Department, King Saud bin Abdulaziz University for Health Sciences, Jeddah, Saudi Arabia; 10 Department of ICU National Hospital, Internal Medicine and Critical Care, Riyadh, Saudi Arabia; 11 Critical Care Medicine Department, Cairo University Hospitals, Cairo, Egypt; 12 Department of Internal Medicine, King Abdulaziz University, Jeddah, Saudi Arabia; 13 Department of Cardiac Surgery, King Abdullah Medical City, Makkah, Saudi Arabia; 14 Department of Surgery/Intensive Care, King Abdulaziz University, Jeddah, Saudi Arabia; 15 Community Medicine Department, Ministry of Health, Jeddah, Saudi Arabia; 16 Department of Medicine, Division of Critical Care, McMaster University, Hamilton, Canada; 17 Department of Critical Care, Prince Sultan Military Medical City, Riyadh, Saudi Arabia; 18 King Abdullah International Medical Research Center, King Saud bin Abdulaziz University for Health Sciences, Riyadh, Saudi Arabia; 19 Interdepartmental Division of Critical Care Medicine, University of Toronto, Toronto, Canada; 20 Department of Clinical Epidemiology and Biostatistics, McMaster University, Hamilton, Canada

**Keywords:** Coronavirus infection, Extracorporeal membrane oxygenation, Rescue therapy, Signs and symptoms respiratory

## Abstract

**Background:**

Middle East respiratory syndrome (MERS) is caused by a coronavirus (MERS‐CoV) and is characterized by hypoxemic respiratory failure. The objective of this study is to compare the outcomes of MERS-CoV patients before and after the availability of extracorporeal membrane oxygenation (ECMO) as a rescue therapy in severely hypoxemic patients who failed conventional strategies.

**Methods:**

We collected data retrospectively on MERS-CoV patients with refractory respiratory failure from April 2014 to December 2015 in 5 intensive care units (ICUs) in Saudi Arabia. Patients were classified into two groups: ECMO versus conventional therapy. Our primary outcome was in-hospital mortality; secondary outcomes included ICU and hospital length of stay.

**Results:**

Thirty-five patients were included; 17 received ECMO and 18 received conventional therapy. Both groups had similar baseline characteristics. The ECMO group had lower in-hospital mortality (65 vs. 100%, *P* = 0.02), longer ICU stay (median 25 vs. 8 days, respectively, *P* < 0.01), and similar hospital stay (median 41 vs. 31 days, *P* = 0.421). In addition, patients in the ECMO group had better PaO2/FiO2 at days 7 and 14 of admission to the ICU (124 vs. 63, and 138 vs. 36, *P* < 0.05), and less use of norepinephrine at days 1 and 14 (29 vs. 80%; and 36 vs. 93%, *P* < 0.05).

**Conclusions:**

ECMO use, as a rescue therapy, was associated with lower mortality in MERS patients with refractory hypoxemia. The results of this, largest to date, support the use of ECMO as a rescue therapy in patients with severe MERS-CoV.

## Background

Middle East respiratory syndrome (MERS), which was first described in 2012, is caused by a novel coronavirus (MERS-CoV). The World Health Organization (WHO) as of 5 December 2016 reported 1917 confirmed cases of the MERS-CoV infection globally with an overall mortality rate of 35% [1]. The majority of cases were reported in Saudi Arabia, wherein 1567 were confirmed cases, and of which 649 (41%) died [2]. Human coronaviruses were first identified in the mid-1960s and usually cause mild upper-respiratory tract illness. In 2012, the first confirmed case of MERS-CoV was reported from Saudi Arabia [3].

MERS-CoV infection is associated with significant mortality related to the virulence of the virus, nature of the disease, and the lack of effective therapy. Patients with MERS-CoV who develop acute respiratory distress syndrome (ARDS) are at a high risk of dying from refractory hypoxemia, multiorgan failure, and septic shock [4].

Current interventions such as lung protective ventilation, prone ventilation, and neuromuscular blocking agents have been shown in randomized trials to improve mortality in patients with ARDS [5–7]. However, in some patients, these conventional measures fail to maintain adequate oxygenation; therefore, other rescue therapies are considered, such as different modes of ventilation, inhaled pulmonary vasodilators, and extracorporeal membrane oxygenation (ECMO). Anticipated difficulties in patient recruitment, study design, and ethical concerns affect the feasibility of conducting randomized clinical trials that examine the efficacy of ECMO in this population. Therefore, observational studies are a reasonable alternative. In this study, we aim to describe the effect of ECMO rescue therapy on patient-important outcomes in patients with severe MERS-CoV.

## Methods

### ECMO program

In response to the large MERS-CoV outbreak, the Saudi Ministry of Health implemented a national ECMO program in April 2014. The Saudi ECMO program provided a rapid transportation chain system (Medevac system), isolated intensive care unit (ICU) beds, and venovenous (V-V) ECMO machines in selected centers across the country. An ECMO team was created that was available 24 h a day/7 days a week. The team included an intensivist trained in ECMO, a cardiac surgeon, a perfusionist, and ECMO-trained nurses. The intensivist on the ECMO team triaged all calls from other centers based on predefined criteria, wherein patients were predetermined to be candidates to receive ECMO or not. Criteria for eligibility to receive ECMO were based on the Extracorporeal Life Support Organization (ELSO) [8] guidelines and are listed below.

### Study design and settings

We retrospectively identified patients who would have been eligible for ECMO but did not receive it because the ECMO program was not available at that time (prior to April 2014). The intervention (ECMO) group was included from five main ECMO centers in three major cities in Saudi Arabia after the program initiation (April 2014 to December 2015). All participating hospitals were accredited by the Joint Commission International and had closed ICUs with 24-h coverage by trained intensivists. We obtained ethics approval from the Saudi Ministry of Health ethics review board and from individual centers’ ethics boards.

### Case definition and ECMO eligibility

Patients were candidates to receive ECMO if they have met the following criteria:

1. Laboratory-confirmed MERS-CoV according to the WHO criteria, which use real-time RT-PCR, assays targeting the up, Orf1a, or Orf1b regions of the MERS-CoV genome from nasopharyngeal swab, tracheal aspirates, or bronchoalveolar lavage (BAL) [9].
2. Were admitted to the ICU and on invasive mechanical ventilation.
3. Met ECMO initiation criteria:
  - Severe respiratory failure defined as a PaO2/FiO2 < 100 on FiO2 > 0.9 and/or
  - Murray score 3–4 despite optimal care for 6 h or more and/or
  - CO_2_ retention on mechanical ventilation despite high P-plat (> 30 cm H_2_O)
4. None of the following contraindications to ECMO:
  - mechanical ventilation at high settings (FiO2 > 0.9, P-plat > 30) for ≥ 7 days
  - recent central nervous system hemorrhage
  - existence of non-recoverable terminal disease

The ECMO group included patients who met the above criteria and received ECMO after implementing the ECMO program from April 2014 to December 2015. We included all patients with MERS-CoV who received ECMO during that period. The control group were patients who met the above criteria but did not receive ECMO in the period prior to the introduction of ECMO program (prior to April 2014). Weaning from ECMO was primarily based on clinical improvement demonstrated by adequate oxygenation and gas exchange shown in vital signs, blood gases, and chest X-ray. The decision for readiness of a patient to be weaned from ECMO was left to the judgment of treating clinician and the ECMO team. The weaning process followed the ELSO criteria as follow: weaning starts by decreasing the flow to 1L/min while keeping the sweep of 100% (to maintain SPO2 > 95%). If SPO2 remains within target, a trial of clamping the catheters and keeping the patient on the ventilator at appropriate settings was attempted.

### Data collection

We designed an electronic pretested data abstraction forms; the forms were pilot tested prior to data collection to ensure accuracy and reproducibility. Trained personnel collected the data at each participating center under the supervision of the local principal investigators. Research personnel collected data on patients’ demographics, comorbidities, Acute Physiology and Chronic Health Evaluation II (APACHE II) score, laboratory results (hemoglobin concentration, white blood and platelets counts, kidney function, blood gases), ventilator modes and settings, interventions used to treat refractory hypoxemia (prone ventilation, use of neuromuscular blocking drugs, and pulmonary vasodilators), vasoactive support, antimicrobial and antiviral therapy, steroid use, and primary and secondary outcome data.

### Statistical analysis

Data were tested for normality using the Kolmogorov–Smirnov test. A repeated-measures analysis of variance was performed. Fischer’s exact test was used for the categorical data. Independent *t* test was used to compare the continuous variables in the two groups. The Mann–Whitney U test was performed to compare the nonparametric values of the two groups. Data were expressed as median (interquartile range (IQR) [range]), number (proportion), or mean (SD) as appropriate. The volume of cases was not enough to allow a priori power analysis. However, a post hoc power analysis indicated that the current sample size of 35 patients is powered to detect 35% absolute difference in mortality rate, with a type I error of 0.05 and a power of 80%. A value of *P* < 0.05 was considered statistically significant.

## Results

### Baseline characteristics

Eighty patients with confirmed MERS-CoV infection were admitted to the ICUs of participating centers from April 2014 to December 2015. Thirty-five patients met our eligibility criteria and were included in the analysis, 17 in the ECMO group and 18 in the control group. As shown in Table 1, the baseline characteristics were similar in both groups; the median ages were (46 vs. 50 years), and mean APACHE II score (28 vs. 31) were not statistically different. (*P* = 0.48 and *P* = 0.12; respectively).

**Table 1:** Patients characteristics

Variable	Group 1 ECMO (*n* = 17)^a^	Group 2 Control (*n* = 18)	*P* value
Age median [IQR]	45.5 [28.5–58.5]	50 [33–63.5]	0.484
Gender (male) *n* (%)	12 (70.6%)	11 (61.1%)	0.556
Weight (kg)	87.4 (25.4)	87.5 (21.4)	0.989
Height (cm)	167.4 (10.1)	161.6 (6.1)	0.100
Body surface area (kg/m^−2^)	1.95 (0.31)	1.90 (0.26)	0.712
APACHE II median [IQR]^b^	27.8 [23–29.8]	31 [24–29.5]	0.120
Pregnancy *n* (%)	1 (5.9%)	1 (5.6%)	0.493
Comorbidities *n* (%)			
Diabetes	8 (47.1%)	10 (55.6%)	0.616
Hypertension	5 (29.1%)	7 (38.9%)	0.725
Coronary artery disease	1 (5.9%)	1 (5.6%)	0.493
Heart failure	0 (0%)	1 (5.6%)	0.975
Bronchial asthma	2 (11.8%)	2 (11.1%)	0.638
COPD^c^	2 (11.8%)	0 (0%)	0.442
Acute kidney injury	2 (11.8%)	1 (5.6%)	0.975
Chronic kidney disease	1 (5.9%)	4 (22.2%)	0.371
Liver disease	0 (0%)	2 (11.1%)	0.493
Immunocompromised	0 (0%)	1 (5.6%)	0.975
Preexisting risk factors for ARDS *n* (%)^d^	0 (0%)	0 (0%)	
Bacterial co-infection *n* (%)	7 (41.2%)	4 (22.2%)	0.401

Data are presented as median [minimum–maximum], number (%), or mean (SD)

^a^*ECMO* extracorporeal membrane oxygenation

^b^*APACHE II* Acute Physiology and Chronic Health Evaluation score II

^c^*COPD* chronic obstructive pulmonary disease

^d^*ARDS* acute respiratory distress syndrome

Adjunctive therapies were used in both groups. Ribavirin was used significantly more often in the ECMO group compared to the control group (82 vs. 24%, *P* = 0.001), interferon was also used more in the ECMO cohort compared to controls (65 vs. 24%, *P* = 0.016), and the use of steroids was similar in both groups (53 vs. 72%, *P* = 0.24). At day one of eligibility to ECMO, more patients in the control group required hemodynamic support with norepinephrine compared to ECMO group; however, both groups had similar use of epinephrine and dobutamine, continuous renal replacement therapy (CRRT), modes of ventilation, positive end-expiratory pressure (PEEP), and neuromuscular blocking agents (Tables 2 and 3). Alveolar recruitment maneuver was used in one patient in the ECMO group. None of the patients received prone ventilation. Throughout days 1–14, more patients in the control group developed renal impairment and had significantly lower PaO2/FiO2 ratio (Table 3). Other laboratory values were similar between both groups (Table 4). However, due to the small sample size, it was not feasible to adjust for all confounding factors.

**Table 2:** Circulatory and renal support during the study period

	Day 1		Day 3		Day 7		Day 10		Day 14	
	ECMO (*n* = 17)	Non-ECMO (*n* = 18)	ECMO (*n* = 14)	Non-ECMO (*n* = 15)	ECMO (*n* = 14)	Non-ECMO (*n* = 15)	ECMO (*n* = 14)	Non-ECMO (*n* = 15)	ECMO (*n* = 14)	Non-ECMO (*n* = 14)
Use of norepinephrine *n* (%)	5 (29.4%)	12 (80%)*	8 (57.1%)	11 (73.3%)	7 (50%)	13 (86.7%)	7 (50%)	12 (80%)	5 (35.7%)	13 (92.9%)*
Max. dose (µg kg^−1^ min^−1^)	0.08 (0.16)	0.13 (0.25)	0.04 (0.55)	0.15 (0.17)*	0.04 (0.58)	0.43 (0.69)*	0.15 (0.30)	0.06 (0.09)	0.44 (0.58)	0.5 (0.71)
Use of dobutamine *n* (%)	2 (11.8%)	2 (11.1%)	0 (0%)	0 (0%)	0 (0%)	0 (0%)	0 (0%)	0 (0%)	0 (0%)	0 (0%)
Max. dose (µg kg^−1^ min^−1^)	1.5 (2.21)	5.0 (8.66)	NA	NA	NA	NA	NA	NA	NA	NA
Use of epinephrine *n* (%)	0 (0%)	NA	0 (0%)	NA	0 (0%)	1 (6.7%)	1 (7.1%)	NA	1 (5.9%)	NA
Max. dose (µg kg^−1^ min^−1^)	NA	NA	NA	NA	NA	1.1 (0.90)	0.10 (0.14)	NA	1.5 (0.00)	NA
Use of CRRT *n* (%)^a^	3 (17.6%)	4 (22.2%)	7 (50%)	6 (40%)	8 (57.1%)	6 (46.7%)	5 (35.7%)	3 (20%)	2 (14.3%)	1 (7.10%)

Results are presented as number (%) or mean (SD)

*P < 0.05 vs. ECMO group

^a^*CRRT* continuous renal replacement therapy

**Table 3:** Ventilatory support data during the study period; data are presented as number (%) or mean (SD)

	Day 1		Day 3		Day 7		Day 10		Day 14	
	ECMO (*n* = 17)	Non-ECMO (*n* = 18)	ECMO (*n* = 14)	Non-ECMO (*n* = 15)	ECMO (*n* = 14)	Non-ECMO (*n* = 15)	ECMO (*n* = 14)	Non-ECMO (*n* = 15)	ECMO (*n* = 14)	Non-ECMO (*n* = 14)
Invasive ventilation *n* (%)	17 (100%)	18 (100%)	14 (100%)	16 (100%)	14 (100%)	15 (100%)	11 (78.6%)	14 (93.3%)	5 (35.7%)	13 (92.9%)*
Mode of ventilation										
CMV	6 (35.3%)	10 (55.6%)	7 (50%)	11 (68.8%)	6 (42.8%)	8 (53.3%)	4 (36.4%)	6 (42.9%)	1 (20%)	11 (84.6%)*
PCV	3 (17.6%)	2 (11.1%)	4 (28.6%)	1 (6.3%)	4 (28.5%)	3 (20%)	3 (27.3%)	5 (35.7%)	3 (60%)	0 (0%)*
VCV	4 (23.5%)	4 (22.2%)	1 (7.1%)	2 (12.5%)	1 (7.1%)	1 (6.7%)	1 (9.1%)	2 (14.3%)	1 (20%)	0 (0%)
PRVC	2 (11.8%)	1 (5.6%)	3 (21.4%)	0 (0%)	1 (7.1%)	2 (13.3%)	0 (0%)	1 (7.1%)	0 (0%)	1 (7.7%)
APRV	1 (6.7%)	1 (5.6%)	1 (7.1%)	0 (0%)	1 (7.1%)	1 (6.7%)	0 (0%)	0 (0%)	0 (0%)	1 (7.7%)
SIMV	1 (5.9%)	0 (0%)	0 (0%)	0 (0%)	0 (0%)	0 (0%)	1 (9.1%)	0 (0%)	0 (0%)	0 (0%)
PSV	0 (0%)	0 (0%)	0 (0%)	0 (0%)	0 (0%)	0 (0%)	1 (9.1%)	0 (0%)	0 (0%)	0 (0%)
CPAP	0 (0%)	0 (0%)	0 (0%)	0 (0%)	0 (0%)	0 (0%)	1 (9.1%)	0 (0%)	0 (0%)	0 (0%)
HFOV	0 (0%)	0 (0%)	0 (0%)	1 (6.3%)	1 (7.1%)	0 (0%)	0 (0%)	0 (0%)	0 (0%)	0 (0%)
FiO_2_ (%)	0.67 (0.29)	0.80 (0.24)	0.59 (0.24)	0.79 (0.25)*	0.50 (0.21)	0.73 (0.24)*	0.50 (0.25)	0.66 (0.27)	0.33 (0.17)	0.72 (0.38)*
PaO_2_/FiO_2_ ratio (%)	115 (110.2)	109 (93.9)	109 (81.51)	63 (62.7)	124 (106.9)	63 (66.1)*	138 (139.5)	36 (66.4)*	237 (42.11)	85 (31.95)*
PEEP (cm H_2_O)	12 (3.97)	12 (6.4)	13 (3.94)	14 (4.8)	11 (3.61)	14 (5.6)	11 (4.04)	11 (5.7)	10 (7.20)	13 (7.0)
Use of NMBs *n* (%)	8 (53.3%)	12 (66.7%)	10 (76.9%)	12 (80%)	10 (71.4)	8 (53.3%)	4 (36.4%)	3 20%)	2 (40%)	0 (0%)

*CMV* continuous mandatory ventilation, *PCV* pressure-controlled ventilation, *VCV* volume-controlled ventilation, *PRVC* pressure-regulatory volume control ventilation, *APRV* airway pressure release ventilation, *SIMV* synchronised intermittent mandatory ventilation, *PSV* pressure-support ventilation, *CPAP* continuous positive airway pressure, *HFOV* high-frequency oscillatory ventilation, *FiO*_*2*_ inspired oxygen fraction, *PaO*_*2*_*/FiO*_*2*_ arterial oxygen tension to FiO_2_ ratio, *PEEP* positive end-expiratory pressure, *ARM* alveolar recruitment maneuver, *NMBs* neuromuscular blocking drugs

*P < 0.05 versus ECMO group

**Table 4:** Laboratory data

	Day 1		Day 3		Day 7		Day 10		Day 14	
	ECMO	Non-ECMO	ECMO	Non-ECMO	ECMO	Non-ECMO	ECMO	Non-ECMO	ECMO	Non-ECMO
Hemoglobin (g dL^−1^)	10.9 (2.44)	10.6 (2.93)	10.3 (1.37)	10.9 (2.31)	9.6 (1.31)	9.9 (1.51)	9.7 (1.29)	8.8 (1.10)	9.3 (0.83)	9.4 (1.10)
WBCs × 10^−9^ L	9.9 (4.05)	12.5 (7.64)	13.9 (7.08)	9.6 (5.42)	15.5 (9.09)	16.7 (14.04)	16.0 (7.23)	28.3 (19.30)	13.3 (10.51)	12.6 (1.10)
Platelets × 10^−9^ L	180 (127.5)	210 (124.9)	149 (87.32)	206 (103.5)	144 (113.7)	195 (101.5)	157 (142.3)	197 (80.1)	183 (158.9)	185 (107.5)
BUN (mg dL^−1^)	36 (25.6)	27 (24.12)	42 (29.1)	29 (23.32)	33 (30.7)	35 (27.69)	43 (34.3)	58 (53.61)	13 (7.2)	37 (32.35)
Creatinine (µmol L^−1^)	205 (149.90)	201 (166.61)	210 (145.69)	254 (153.75)	140 (88.0)	223 (132.83)	160 (100.9)	295 (182.23)	74 (34.40)	289 (133.75)*
Arterial blood gases										
pH	7.39 (0.07)	7.24 (0.19)*	7.35 (0.11)	7.28 (0.13)	7.35 (0.06)	7.24 (0.15)*	7.34 (0.09)	7.27 (0.14)	7.29 (0.22)	7.32 (0.01)
PaCO_2_ (mmHg)	38.8 (8.88)	44.5 (14.71)	41.5 (8.94)	53.2 (19.21)*	41.7 (5.77)	57.8 (20.51)*	44.5(17.91)	48.1 (11.81)	60.7 (45.08)	53.7 (20.93)
HCO_3_ (mEq L^−1^)	23.2 (4.11)	21.5 (7.01)	23.3 (5.16)	23.4 (2.61)	24.2 (4.71)	22.9 (6.12)	22.0 (4.36)	22.1 (4.41)	23.6 (6.98)	26.0 (3.31)
PaO_2_ (mmHg)	83.7 (42.48)	92.7 (76.02)	71.1 (16.61)	58.7 (18.00)	81.9 (44.52)	66.1 (20.81)	92.1 (37.13)	79.1 (24.71)	78.2 (41.21)	61.0 (32.16)

Data are presented as mean (SD)

*ECMO* extracorporeal membrane oxygenation, *WBCs* white blood cells, *BUN* blood urea nitrogen, *PaCO*_*2*_ arterial carbon dioxide tension, *HCO*_*3*_ bicarbonate, *PaO*_*2*_ arterial oxygen tension

^*^P < 0.05 versus ECMO group

### The intervention

In the ECMO group, the V-V mode was used in all patients via the percutaneous cannulation approach for vascular access. Femoral–femoral access was used in 65% of patients, while femoral–jugular access was used in 35% of cases. ECMO access was inserted by a cardiac surgeon in 70% of cases and by a cardiac intensivist in the remaining 30%. Chest X-ray was used to confirm successful cannulation in 16 patients and transesophageal echocardiography (TEE) in one patient. Blood flow (L min^−1^), revolutions per minute, and sweep gas among ECMO patients had a mean (SD) of 3.8 (0.77), 3148.7 (933.8), and 21.6 (63.2), respectively. Patients who were in the same city, where ECMO centers were situated, were transported via ground ambulances; otherwise, patients were transported via fixed wing medical evacuation airplane. No complication was reported during patients’ transportation.

ECMO-related mechanical complications occurred in 3 (18%) patients; one patient developed pneumothorax that was treated with chest tube insertion, and two patients had major bleeding immediately after the initiation of ECMO.

### Outcome

**Fig. 1:**
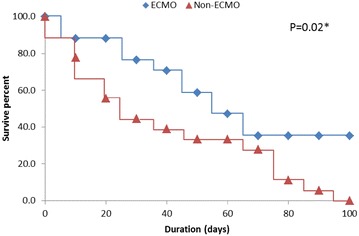
Kaplan–Meier survival curves of the two studied groups. *P* value for log-rank test is shown

Compared to the control group, the ECMO group had significantly lower in-hospital mortality (65 vs. 100%; *P* = 0.02), longer ICU stay (25 vs. 8 days; *P* = 0.001) (Table 5 and Fig. 1). Less use of norepinephrine at days 1 and 14 (*P* < 0.05), and better oxygenation (higher PaO_2_/FiO_2_ ratio) throughout days 7–14 (Table 2).

**Table 5:** Outcomes of patients treated with ECMO compared to patients managed without ECMO

Variable	ECMO (*n* = 17)	Control (*n* = 18)	*P* value
In-hospital mortality *n* (%)	11 (64.7%)	18 (100%)	0.020
ICU length of stay (days)^a^	22.5 [12.5–28.3]	7 [4.3–11.5]	0.001
Hospital length of stay (days)	25 [6.3–56.5]	47 [5–76.5]	0.421
Time to death (days)	32 [1–68]	47 [1-93]	0.422
Cause of death *n* (%)			
Septic shock/infection	7 (41.2%)	4 (22.2%)	0.401
Refractory hypoxemia	3 (17.6%)	2 (11.1%)	0.975
Others (undetermined)	1 (5.9%)	12 (66.7%)	0.042

Data are presented as number (%), mean (SD), or median [minimum–maximum]

^a^*ICU* intensive care unit

## Discussion

In this retrospective cohort study, we found that ECMO rescue therapy was associated with lower in-hospital mortality, better oxygenation, and fewer organ failures compared to historical control (usual care) in patients with severe MERS-CoV. However, the length of hospital stay was the same and a possible explanation is that during the crisis phase, patients were mechanically ventilated in the ward when ICU beds are full, and it is possible that this could have contributed to similar stay in hospital in both groups.

Although ELSO issued guidelines on the use of ECMO in patients with ARDS, these guidelines do not address specific disease context, and are difficult to generalize to the heterogeneous ARDS population. Therefore, we conducted this observational study to report on the efficacy and safety of ECMO in patients with severe MERS-CoV infection.

There is a single case report in the literature looking at ECMO in MERS-CoV patients. Guery et al. described the use of ECMO in two patients with acute respiratory failure secondary to MERS-CoV infection in France, where both patients developed severe hypoxia and increasing oxygen requirements, leading to mechanical ventilation and ECMO use. One patient died, and the other survived after approximately 2 months in hospital [10].

ECMO use in respiratory failure has been reported with variable survival rates. The first 2 randomized clinical trials (RCTs) failed to prove superiority of ECMO over conventional management [11, 12]. However, the severe adult respiratory failure (CESAR) trial showed improved 6-month survival in patients who were referred early to an ECMO center [13]. This was the largest clinical trial to investigate the efficacy of early use ECMO in patients with ARDS. Despite concerns about the trial design and possible differences in steroid use and ventilator strategies, these results contributed to the increasing use of ECMO worldwide.

In this study, we observed no significant differences in the use of adjunctive therapies except for ribavirin use in the ECMO group. The benefit of antiviral therapy in MERS-CoV infection remains unclear. Recent Korean guidelines published during the 2015 MERS-CoV outbreak in South Korea suggested the use of antiviral therapy in patients with severe MERS-CoV [14].

In patients with respiratory failure from H1N1 infection who required the use of ECMO, the survival rate varied considerably between studies ranging from 35 to 90% [15–28]. There was a large variation in survival rates, which could be explained by differences in patients’ baseline characteristics and severity of illness. In one study, older, obese, diabetic, or immunocompromised patients were found to be at a higher risk of developing severe MERS-CoV infection [28–31]. In this study, the two groups were comparable at baseline, and there were no significant differences between groups in any of these variables. Another large observational study examined the predictors of death in H1N1 patients who underwent V–V ECMO and found that creatinine and bilirubin levels, systemic arterial pressure, hematocrit, and pre-ECMO hospital length of stay were associated with higher mortality [32].

Another important factor is the center experience and volume of cases; this could have contributed to the variability in survival rates with ECMO use. A recent study by Barbaro et al. [33] demonstrated that centers with > 30 ECMO cases/year had better survival rates than centers with less than 6 cases per year. In Saudi Arabia, ECMO was not available except in one center until the MERS-CoV crisis; thereafter, the ECMO program was implemented as a therapeutic option for patients with refractory hypoxemia. ECMO interventions were run in tertiary centers with equipped ICUs by most experienced intensivists and perfusionists who received training in ECMO prior to the start of the program.

Although more ECMO patients received ribavirin and interferon therapy, we do not believe that this difference has an impact to our findings. Published reports on this therapy are limited, but none showed significant improvement with this combination [34–36]. The largest study to date published in abstract format [37] showed no reduction in mortality. Therefore, we believe that the imbalance of co-interventions between the two groups is unlikely to affect the estimation of treatment effect.

In regard to infection control issues, caregivers safety of ECMO patients was organized and maintained by aggressive measures which were applied strictly and monitored closely with all admissions were taken to airborne isolated rooms which impacted the containment of the virus plus applying the universal protective personal measures all the time during the patients encounter. Because of these stringent measures, there were no reports by or about any caregiver of any ECMO patient being affected.

To our knowledge, this is the largest study to describe outcomes in patients with MERS-CoV who received ECMO. There are several strengths to our study: the “before and after” design allowed us to compare ECMO cases to a control group with similar demographics and within the same institutions. We also collected data on important variables and confounders, and conducted adjusted analyses to assess the impact on the results. We adhered to the Strengthening the Reporting of Observational studies in Epidemiology (STROBE) guidelines [38].

Despite the strengths of our study, it has several important limitations. First, the retrospective nature of this study renders it at risk of bias. All patients in the control group died, which may be explained by the severity of illness, as these were patients who had ARDS and were eligible otherwise. We cannot rule out the possibility of selection bias, as we were unable to track all transfer requests due to the outbreak and crisis at the time, leaving us with limited information. In addition, some patients were transferred from non-participating ECMO centers; therefore, baseline pre-ECMO data such as blood gases and ventilator settings could not be obtained. Furthermore, due to insufficient documentation during the outbreak and crisis circumstances, we were not able to track the ECMO requests to the referral call center.

There were differences in some co-interventions (e.g., antiviral therapy), and the influence of unmeasured confounders cannot be excluded. Such concerns can only be addressed in RCTs; however, conducting RCT is likely to be challenging in the context of epidemics. This study was not designed to compare the cost of 2 interventions; although it is an important outcome that could help the clinicians and stakeholders to make decisions. Lastly, the small sample size limited our ability to perform an adequate multivariate analysis. Similar to other ECMO studies, it is difficult to determine if the mortality was the result of refractory respiratory failure or other causes like septic shock or other organs failure.

In summary, the use of ECMO was associated with lower mortality in patients with severe MERS-CoV infection and refractory hypoxia. Future randomized trials, although challenging to conduct, are highly needed to confirm or dispute these observations. Until more data are available, ECMO could be considered as a rescue therapy in selected MERS-CoV patients with refractory hypoxemia.

## Abbreviations

APACHE II: Acute Physiology and Chronic Health Evaluation II
ARDS: acute respiratory distress syndrome
CRRT: continuous renal replacement therapy
ECMO: extracorporeal membrane oxygenation
ELSO: Extracorporeal Life Support Organization
ICU: intensive care units
MERS: Middle East respiratory syndrome
MERS‐CoV: Middle East respiratory syndrome coronavirus
PEEP: positive end-expiratory pressure
RCT: randomized clinical trials
TEE: transesophageal echocardiography
WHO: World Health Organization
